# Coronavirus Disease (Covid-19): What Are We Learning in a Country With High Mortality Rate?

**DOI:** 10.3389/fimmu.2020.01208

**Published:** 2020-05-28

**Authors:** Luciano Mutti, Francesca Pentimalli, Giovanni Baglio, Patrizia Maiorano, Rita Emilena Saladino, Pierpaolo Correale, Antonio Giordano

**Affiliations:** ^1^Center for Biotechnology, Sbarro Institute for Cancer Research and Molecular Medicine, College of Science and Technology, Temple University, Philadelphia, PA, United States; ^2^Cell Biology and Biotherapy Unit, Istituto Nazionale Tumori, IRCCS, Fondazione G. Pascale, Naples, Italy; ^3^Ministry of Health, Rome, Italy; ^4^Department of Medical Biotechnologies, University of Siena, Siena, Italy; ^5^Tissue Typing Unit, Grand Metropolitan Hospital “Bianchi Melacrino Morelli”, Reggio Calabria, Italy; ^6^Unit of Medical Oncology, Oncology Department, Grand Metropolitan Hospital “Bianchi Melacrino Morelli”, Reggio Calabria, Italy

**Keywords:** SARS-CoV2, coronavirus, COVID-19, HLA, ARDS (acute respiratory distress syndrome)

COVID-19 has been declared a pandemic by the WHO (1). Following the outbreak of the disease in China, Italy was the first European country to be heavily struck (2, 3). Initially, three COVID-19 cases were reported in early February, which were all related to individuals who had traveled to China; then, on the 20th, a young man who had not traveled abroad presented with severe SARS-CoV-2-induced pneumonia in Lombardy, a region in the North of the country (2). Over the next 2 weeks, many patients in the surrounding areas were diagnosed with COVID-19, which was often severe, and another cluster was identified in the nearby region of Veneto (2). There then followed an exponential increase in cases, mostly in the North, although the disease spread throughout the whole country, leading to the hypothesis that the virus had been circulating since January (2, 4). At that point, Italy reached incidence and mortality rates that were amongst the highest in the world (2–4). Many factors explain differences from other countries, including different application of detection tests, a larger elderly population, and different prevention policies and capacity to provide intensive care (2). While it is paramount to conceive preventive strategies and apply more effective early treatments, it is also crucial to understand the biological mechanisms underlying these fatal outcomes.

In Italy, the possibility of performing autopsies or post-mortem diagnostic studies on suspect, probable, or confirmed COVID-19 cases has been intensively debated (5, 6); however, post-mortem pathological analysis of COVID-19 patients in China has shown findings consistent with Acute Respiratory Distress Syndrome (ARDS) (7–9) (Figure 1). At present, the exact nature of the acute lung injury trigger is not yet fully clarified; however, it could be ignited by T cells overreacting to virus-specific epitopes, thus recruiting multiple cytokine-activated inflammatory cell lineages (10–12). Other possibilities that deserve further experimental evidence include an exaggerated antibody-mediated response with complement activation and/or FCγ1 receptor-mediated leukocyte engagement and/or a hypothetical cytopathic effect of the virus (13, 14). The latter could explain the recently described microvascular damage leading to disseminated intravascular coagulation (manifested as thrombosis, thrombocytopenia, and gangrene of extremities), anti-phospholipid syndrome, and mimicry of vasculitis, which are described in both Chinese cohorts (15) and Italian patients (16–18).

**Figure 1:**
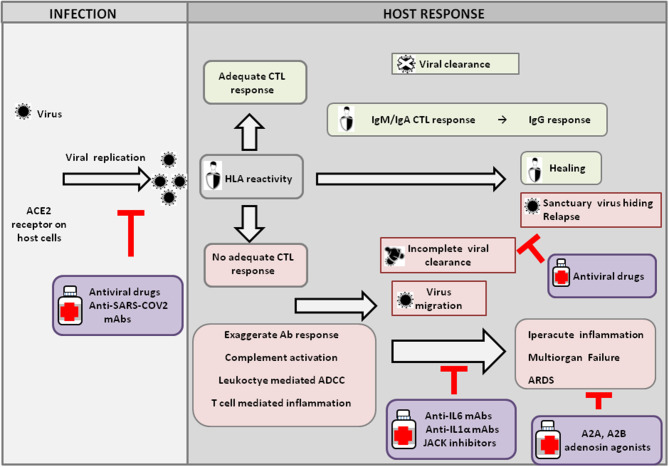
Host response and possible outcomes of SARS-CoV-2 infection. Viral infection seems to occur mainly upon SARS-CoV2 engagement of angiotensin I converting enzyme 2 (ACE2), which acts as a functional receptor for the spike glycoprotein of the coronavirus. The HLA genetic system acts as a key player in determining the anti-viral immune response. In particular, the ability of HLA to trigger an adequate cytotoxic T-lymphocyte (CTL) response will result in viral clearance and host healing, along with the development of the IgM, IgA, and IgG humoral response. Conversely, an inadequate HLA asset will result in an inefficient CTL response and, consequently, incomplete viral clearance. In this context, various factors underlie increased COVID-19 severity, including an exaggerated Ab response, complement activation, leukocyte-mediated antibody-dependent cell-mediated cytotoxicity (ADCC), and T-cell-mediated inflammation, as discussed in the text. Without a protective immune response, the virus is able to migrate, propagating into other ACE2-expressing tissues, while the damaged lung cells induce high inflammation, triggering the cytokine storm that represents the main cause of the acute respiratory distress syndrome (ARDS) and subsequent multiorgan failure. Incomplete viral clearance can also lead to virus hiding in sanctuary sites and patient relapse with symptoms arising in new districts. In the purple boxes, different therapeutic approaches aimed at targeting either the virus or endogenous host players are represented.

In our experience, ≈18% of patients develop interstitial pneumonia, and a subset of these (≈5%) develop ARDS that, especially when so serious as to require invasive ventilation, is mostly fatal. The risk of ARDS rises with age, and almost all deaths regard patients with pre-existing chronic conditions (19, 20). Pre-admission hypertension, in particular, has been reported as a key mortality risk factor (19). The risk of death further rises where there is a lack of ventilators or ventilation is refused, as described in Xu et al. (9).

Moreover, an increasing number of clinical reports describe a biphasic behavior: a first phase where COVID-19-infected patients are completely asymptomatic, which lasts on average seven days, and a second phase where the patients present mild to moderate flu-like symptoms, anosmia, ageusia, and blind conjunctivitis, which may last 10–15 days (21, 22). A minority of patients who are unable to achieve complete virus coverage develop severe cardio-respiratory symptoms with radiological signs of pneumonitis, ARDS, and then multiorgan failure (23). The last phase occurs, on average, 15–30 days after infection. In the latter case, patients may test negative for COVID genome research standard molecular tests. Altogether, these clinical findings, as well as the available pathology studies, support the hypothesis of an inappropriate immune-related inflammatory response to COVID19 epitopes and consequent auto-antigen release and T-cell cross-presentation in the damaged alveolar tissue. Consistently, recent results indicate that a systemic immune dysregulation that triggers auto-sustaining inflammatory lung damage, causing fatal respiratory-failure and consequent multiorgan-failure, is the main virus-related-death cause in patients who develop SARS-CoV-2 (10).

The culprit is the *cytokine storm* unleashed in this context by the infection and already described in cancer patients treated with CART or immunotherapy, including the “old” treatments with interleukins (IL2 and IL12, in particular) and the newest anti-CTLA-4 and or anti-PD-1/PDL1 immune-checkpoint inhibitors. A greater risk of pneumonitis has already been recorded in Chinese patients bearing a high-frequency of specific class-I and II HLA alleles associated with poor virus clearance and development of immune-related pneumonitis and other inflammatory-related autoimmune diseases (24).

This viral-load-independent different response to the infection might depend on a genetic predisposition causing extreme and often lethal inflammatory reactions.

Given the inefficacy of steroids (9), understanding the molecular features underlying such threatening immune-related events provides a strong rationale for using biological drugs for the early treatment of symptomatic patients, aimed at hampering the effects of the most relevant cytokines able to trigger an antibody response and acute inflammatory reaction, such as IL6 and IL1α. To this purpose, Abs against the IL6 receptor, or drugs able to disrupt its downstream signals, can inhibit its function on specific inflammatory cell subsets. These agents have so far been promising in the clinical setting for curbing the inflammatory response to control the severe immune-related adverse events related to CART-therapy and immune-checkpoint blockade and autoimmune diseases, including Juvenile Rheumatoid Arthritis, Psoriatic Arthritis, and Ulcerative colitis, all related to particular HLA Class I and II alleles, some of which, like class I B^*^27 and B^*^35, might sustain both mitochondrial stress and cross-reactivity with several pathogens (25).

Therefore, while antiviral drugs help to contain viral replication, moAbs to IL6 in the early phase of respiratory involvement could control the risk of a fatal virus-induced-cytokine storm. A great effort should be made to recognize lung involvement as, at least theoretically, the earlier the treatment, the better the outcome will be, with IL6 inhibitors being able to “nip in the bud” the inflammatory cascade and prevent the fatal permanent damage to the alveolar pneumocytes. On this basis, IL6 inhibitors are currently being tested in China and Italy in patients with respiratory failure, and other IL6 inhibitors are also being considered.

Iatrogenic cues might also contribute to exacerbating the acute inflammatory lung injury triggered by the virus. Most hospitalized patients in fact received oxygen either through intubation or mechanical or non-invasive ventilation (20); however, oxygenation in ARDS patients with acute lung inflammation has been previously shown to interfere with the anti-inflammatory response induced locally by hypoxia through the activation of the adenosine A2A receptor (26). Similarly, in COVID-19, patients, oxygen therapy could worsen lung injury by weakening such anti-inflammatory pathways. Consistently with this hypothesis, in a cohort of 5,700 patients hospitalized with COVID-19 in the New York City area, mortality reached 88.1% for those requiring mechanical ventilation (27). In Lombardy, the intensive care unit mortality was 26%, and indeed, a large proportion of admitted patients required mechanical ventilation (20). These data support the possible use of adenosine agonists in patients presenting with ARDS (Figure 1).

Identifying infected patients at higher risk of poor prognosis even without evident risk factors could represent an important step forward. In this direction, Zhou et al. reported some predictive biomarkers of the severity of the infection (23). Nguyen and colleagues, in a preprint article, analyzed the SARS-CoV-2 proteome and identified a range of HLA alleles potentially able to present (or not) viral epitopes. They suggest that individuals bearing HLA-B^*^46 (which has the fewest predicted binding peptides for SARS-CoV-2) may be particularly vulnerable to COVID-19, whereas individuals bearing HLA-B^*^15 (which has the greatest predicted capacity to present SARS-CoV-2 peptides) could exhibit cross-protective T-cell based immunity. The authors highlight that a thorough understanding of how HLA variation correlates with COVID-19 onset and outcome could help identify high-risk subjects (28). Indeed, we have preliminary evidence that the prevalence of specific HLA class I alleles across Italian regions/provinces correlates with increased COVID-19 incidence (Correale P., Mutti L., submitted for publication). If confirmed in wide case-control studies, the identification of HLA alleles that are more permissive to viral infection would provide the first genetic explanation for the wide differences in COVID-19 incidence rates among Italian regions and also among nearby provinces with similar environmental factors.

Overall, understanding the role of pro-inflammatory cytokines certainly unravels a new battleground against the lethal clinical effect of CODIV-19 infection; this, along with the identification of a high-risk autoimmune profile, including the genotyping of Class I and II HLA, which have a key role in shaping the anti-viral immune response and Th1/Th2 lymphocyte subset response (Figure 1), and immune-profiling, could also help to prevent these dangerous evolutions of the disease (29). In particular, the isolation of genetically at-risk individuals, including healthcare workers, will inform future vaccination campaign priorities and clinical management strategies.

The finding of healed patients retesting positive after an apparent complete virus clearance is a matter of intense debate in Italy and worldwide. Assuming that testing was reliable, various hypotheses are being considered, including viral mutation, although variation among sequences seems very low at present (30). A preprint study in rhesus macaques argues against a risk of re-infection (31). Host inability to develop immunological memory with subsequent long-term protection is also being evaluated. Interestingly, another preprint study identified specific SARS-COV-2 neutralizing antibodies (NAbs) in the plasma of patients who had recovered from infection and recorded that 30% of patients failed to develop high titers of NAbs after COVID-19 infection (32). Another possibility is that newborn SARS-CoV-2 might hide in sanctuary sites, such as the NCS and/or testis, which are protected from both antiviral drugs and proficient immuno-effectors; this hypothesis is supported by the recent description of viral detection in the cerebrospinal fluid but not in the nasopharyngeal swab in a case report (33).

Overall, these distinct biological patterns of response to the virus should be taken into account for the design of new preventive and therapeutic strategies.

## Author Contributions

LM, PC, and AG conceived the study. FP and GB evaluated current data. RS and PM studied HLA involvement. PC conceived and FP sketched the figure. All authors contributed to manuscript writing and agreed with content.

## Conflict of Interest

The authors declare that the research was conducted in the absence of any commercial or financial relationships that could be construed as a potential conflict of interest.
